# Correction to: GIL: a python package for designing custom indexing primers

**DOI:** 10.1093/bioinformatics/btad735

**Published:** 2023-12-09

This is a correction to: Nicholas Mateyko, Omar Tariq, Xinyi E Chen, Will Cheney, Asfar Lathif Salaudeen, Ishika Luthra, Najmeh Nikpour, Abdul Muntakim Rafi, Hadis Kamali Deghan, Cassandra Jensen, Carl de Boer, GIL: a python package for designing custom indexing primers, *Bioinformatics*, Volume 39, Issue 6, June 2023, btad328, https://doi.org/10.1093/bioinformatics/btad328

In the originally published version of this manuscript, a co-author’s name was incorrect. The name was incorrectly spelt Hadis Kamali Deghan instead of the correct version “Hadis Kamali Dehghan”.

This error has been corrected online.

